# Toronto Staging Guidelines for Wilms Tumour: The Meeting Point Between Clinicians and Epidemiologists—Results of the BENCHISTA-ITA Project

**DOI:** 10.3390/cancers18132111

**Published:** 2026-06-29

**Authors:** Laura Botta, Fabio Didonè, Riccardo Capocaccia, Massimo Conte, Marcella Sessa, Fabio Savoia, Andrea Di Cataldo, Marta Arrabito, Milena Maria Maule, Gemma Gatta, Rosalia Ragusa, The BENCHISTA-ITA WG

**Affiliations:** 1 Evaluative Epidemiology Unit, Department of Epidemiology and Data Science, Fondazione IRCCS Istituto Nazionale dei Tumori, 20133 Milan, Italy; fabio.didone@istitutotumori.mi.it; 2 Editorial Board, Epidemiologia & Prevenzione, 20133 Milan, Italy; capocaccia.riccardo@gmail.com; 3 Clinical Oncology Unit, IRCCS Istituto Giannina Gaslini, 16147 Genoa, Italy; massimoconte@gaslini.org; 4 Epidemiology, Biostatistics and Childhood Cancer Registry Unit, Santobono-Pausilipon Children’s Hospital, 80129 Napoli, Italy; m.sessa@santobonopausilipon.it (M.S.); f.savoia@santobonopausilipon.it (F.S.); 5 Unit of Pediatric Hematology and Oncology, Azienda Ospedaliero Universitaria Policlinico, 95123 Catania, Italy; adicata@unict.it (A.D.C.); marta-arrabito@hotmail.it (M.A.); 6 Childhood Cancer Registry of Piedmont, Cancer Epidemiology Unit, Department of Medical Sciences, University of Turin and CPO-Piemonte, AOU Città della Salute e della Scienza di Torino, 10126 Turin, Italy; milena.maule@unito.it; 7 Department of Statistics and Quantitative Methods, University of Milano-Bicocca, 20148 Milan, Italy; gemma.gatta@unimib.it; 8 Catania-Messina-Enna Cancer Registry, Azienda Ospedaliero Universitaria Policlinico, 95123 Catania, Italy; rosaliamaria.ragusa@unict.it

**Keywords:** Wilms tumour, stage at diagnosis, survival, population-based cancer registry, children, hospital migration

## Abstract

Background/Objectives: Despite overall excellent outcomes for Wilms tumour, regional variations in stage at diagnosis and care pathways remain a concern across Europe. We evaluated stage distribution, three-year survival, and treatment patterns in Italy, considering hospital care as a proxy for healthcare capacity and migration. Methods: Data were obtained from 26 population-based cancer registries (PBCRs), covering 148 patients (ages 0–14) diagnosed between 2013 and 2017, representing about 80% of the Italian population. Stage was classified according to the Toronto guidelines. Information on treatment and diagnosed/treating hospitals was collected. Stage at diagnosis was further refined using probabilistic linkage with the clinical registry 1.01 Model. Overall survival, defined as all-cause mortality, was estimated using the Kaplan–Meier method. Results: Most patients presented with localized disease (77%), 32% Stage I, while 19% were Stage IV. Three-year survival analysis showed significant differences between stages, ranging from 98% in patients with Stage I to 78% in the ones with Stage IV. No significant disparity across the Italian regions was observed in stage distribution or survival. Diagnoses and treatments were mostly (>90%) centralized in the same region for patients residing in the Centre or North of Italy. However, the cross-regional health migration from the South was of about 30% for diagnosis and larger for treatments. Conclusions: This study shows that standardized staging improves data comparability and highlights challenges in managing metastatic cases and regional care pathways. The results support the use of clinical and PBCR information to interpret survival patterns and guide improvements in paediatric oncology care.

## Simple Summary

This population-based study provided updated figures on Wilms tumour in Italian children. We evaluated three-year survival according to disease stage at diagnosis and explored whether patients moving outside their home region for treatment influenced outcomes. Using standardized data from a large national project BENCHISTA-ITA, we observed high overall survival (~90%), with clear differences by stage, as expected for this tumour. Survival did not vary significantly among Italian macro-areas, but some differences were observed. Patient migration from southern regions was moderate. Overall, our findings highlight the strength of the Italian national clinical network in ensuring consistent high-quality care for children with Wilms tumours. The study also reinforces the importance of collecting information on stage at diagnosis in population-based registries to support continuous improvements in paediatric oncology care.

## 1. Introduction

Wilms tumour (WT), or nephroblastoma, is the most common malignant renal tumour in childhood, predominantly affecting children under five years of age and accounting for approximately 80–90% of all paediatric renal cancers [1,2,3]. In Italy, age standardised incidence rates, based on the European standard population, are approximately 9 cases per million children per year [4,5], slightly lower than those reported in other European regions [6].

WT is considered a major success story in paediatric oncology, owing to the high survival rates achieved through standardized multimodal treatment protocols. Nevertheless, notable disparities in outcomes persist across Europe, often related to differences in tumour stage at diagnosis, access to specialised care, and heterogeneity in treatment approaches [7]. In Italy, survival remains high (89.5%) with relatively small differences across regions and over time [8]. However, these estimates do not account for stage at diagnosis, a key prognostic factor that has not been consistently available or standardized in the studies mentioned above.

To better understand and reduce international disparities, the BENCHISTA (Benchmarking Childhood Cancer Survival by Stage at Diagnosis) project was initiated as a large collaborative effort involving more than 70 population-based cancer registries (PBCRs) worldwide [9]. The project adopts the Toronto Childhood Cancer Stage Guidelines [10,11], a consensus-based system designed to harmonize the collection of staging information for paediatric cancers across PBCRs. These guidelines aim to improve the comparability of stage at diagnosis and a limited set of key non-stage prognostic factors (NSP), thereby strengthening the investigation of survival differences and supporting international benchmarking initiatives.

In Italy, the BENCHISTA-ITA initiative was established to implement these guidelines at the national level [12], supported by structured training, quality assurance procedures, and active collaboration among cancer registries, paediatric oncologists, and pathologists. Compared with the international BENCHISTA project, the Italian initiative collected data on three additional tumour types and included detailed information on hospital location across the different phases of care. Furthermore, it aims to provide more granular insights into within-country variability by conducting analyses at the subnational level, based on Italian macro-areas.

This paper updates the epidemiological evidence on WT in Italy, focusing on children aged 0–14 years, using the data provided by the BENCHISTA-ITA network. More specifically, we analysed stage distribution, survival outcomes, and treatment patterns across three Italian macro-areas of residence at diagnosis (North, Centre, and South and Islands). In addition, information on the hospital where diagnosis and treatment were performed was used as a proxy to investigate regional healthcare capacity and patient mobility.

## 2. Materials and Methods

The study population consisted of all patients under 15 years of age diagnosed with primary WT between 1 January 2013, and 31 December 2017 in Italy, based on data from PBCRs contributing at least three years of incidence within this period. Participating PBCRs were required to provide at least three consecutive years of incidence data while ensuring a minimum follow-up of three years. Vital status in the Italian PBCRs is routinely assessed through linkage with official population registries, (e.g., municipal population registries, National registry of cause of death-RENCAM).

Cancer cases were provided by the 26 PBCRs participating in the BENCHISTA-ITA project and accredited by the Italian Association of Cancer Registries (AIRTUM), collectively covering 84% of the Italian population.

Tumours were classified according to the International Classification of Childhood Cancer, Third Edition (ICCC-3), specifically group VIa, which includes ICD-O morphology codes 8959 and 8960 and topography code C64.9.

To increase the completeness of clinical variables and compare incidence patterns, PBCR data were matched through probabilistic linkage [13] with the Italian Association for Paediatric Haematology Oncology (AIEOP) clinical registry 1.01 Model [14].

Staging at diagnosis was defined according to the Toronto Guidelines (TGs) [15]. Two major clinical protocols were adopted in Italy, each producing a distinct staging system, but associated with comparable survival outcomes [16]. For children treated according to the Children’s Oncology Group/National Wilms Tumour Study Group (COG) protocol, staging was based on the surgical specimen (nephrectomy), as no chemotherapy is administered before surgery. In contrast, patients treated following the International Society of Paediatric Oncology (SIOP) protocol were staged based on surgical findings after receiving neo-adjuvant chemotherapy. Both protocols recognize metastatic disease (Stage IV) at diagnosis based on imaging (Supplementary Table S1) [17].

Where detailed clinical information was available, the more granular Tier 2 TG system was applied [11]. Registries were encouraged to document the clinical sources used for staging, including imaging techniques, such as abdominal ultrasound, CT scans, and MRI. These variables were formally requested and included in the record structure [12].

To ensure consistent interpretation and application of the TG, the BENCHISTA-ITA, in coordination with the international BENCHISTA project, developed a structured training programme. This included in-person workshops and virtual sessions led by paediatric oncology experts, involving staff from PBCRs, paediatric oncologists, and pathologists. Staging data quality was further supported through case-based exercises and a dedicated helpdesk [15].

### Statistical Analysis

A descriptive statistical analysis was performed to summarize tumour stage distribution and to assess survival outcomes across three Italian macro-areas of residence at diagnosis (North, Centre, South and Islands). Pearson’s chi-square test was used to evaluate differences in stage distribution across areas.

Overall survival (OS), defined as the time from diagnosis to death from any cause, was estimated using the Kaplan–Meier method. Survival curves were stratified by TG stage and macro-area. Differences in survival distributions were assessed using the log-rank test. A multivariable Cox proportional hazards model was applied to evaluate the association between stage, age group, macro-area, and migration status, and OS.

To investigate the patterns in healthcare access, we assessed the proportion of children diagnosed and treated within their region of residence. For the purpose of the multivariable analysis, migration was defined as receiving diagnosis and/or at least one treatment outside the region of residence. This binary classification was adopted as a pragmatic approach, given the limited sample size and the rarity of the disease, which did not allow for a more detailed characterization of patient mobility patterns.

All statistical analyses were performed using STATA 17.

## 3. Results

### 3.1. Patient Characteristics and Stage Distribution

A total of 148 incident cases of WT met the inclusion criteria. Cases diagnosed in 2013–2017 were drawn from PBCRs providing at least three years of incidence data within this timeframe. (Table 1). Most cases were registered in the north (45%), followed by the south (34%) and the centre (21%). Basilicata and North Sardinia PBCRs did not contribute with cases, as no Wilms tumour was diagnosed in these regions during the study period.

Information on tumour laterality was available for 136 patients (92%); among these, only 6 presented with bilateral disease. The mean age at diagnosis was 44 months (range: 33–48 months). The presence or absence of anaplasia, the only non-stage prognostic factor required for WT according to the TG, was available for 70% of cases (Table 1). Anaplasia was identified in 15% of patients with available information: in three cases, the subtype (focal versus diffuse) could not be determined, while four cases were classified as focal, and nine as diffuse anaplasia (Supplementary Table S2).

Sixty-one percent of children received chemotherapy prior to surgery, with proportions reaching up to sixty-nine percent across the Italian areas. Loss to follow-up was minimal across all macro-areas.

The linkage with the hospital-based paediatric cancer registry correctly linked 125 cases out of 148, of which 13 benefited from improved staging information from the AIEOP database.

The distribution of tumour stage according to TG Tier 1 and Tier 2, by macro-area, is presented in Table 2. Based on the less granular Tier 1 definition, 77% of cases were classified as localized (L), 22% as metastatic (M), and 1% (2 cases) as unknown. As shown in Table 2, the proportion of metastatic cases was comparable across the three Italian macro-areas.

Overall, Stage I accounted for 32% of cases and was most frequent in the north (36%), followed by the centre (33%) and the south (26%). Stage II represented 23% of cases ranging from 17% in the centre to 27% in the south. Stages III (16% overall) and IV (19% overall) were similarly distributed across regions. The proportion of cases with unknown Tier 2 stage was 3% in the north, 7.8% in the south and 33% in the centre.

### 3.2. Treatment and Region of Treatment

Among the 132 cases for which treatment information was available, all patients underwent surgery, and surgery was combined with chemotherapy and/or radiotherapy in all but four cases (Supplementary Table S3). Regarding treatment patterns, 68% of patients received chemotherapy and surgery, 27% were treated with all three modalities (including radiotherapy), and 3% underwent surgery alone. Information on the hospital of diagnosis and treatment was available for 136 patients (92%). Table 3 reports the proportion of patients who were diagnosed/treated within their region of residence across different phases of care (diagnosis, surgery, chemotherapy, and radiotherapy). In Northern Italy (Veneto, Lombardy, Friuli Venezia Giulia, Trentino-Alto Adige, Liguria, Piedmont, Emilia-Romagna), 92% of children were diagnosed within their region of residence, compared with 72% of those residing in the South (Campania, Apulia, and Sicily). All children living in the central regions (Lazio, Umbria, Marche) were diagnosed within their region of residence (Table 3). In the North, 90% of patients received both surgery and chemotherapy within their region of residence, and radiotherapy was also predominantly administered locally (95%). However, the cancer registry of Tuscany was unable to provide data on treatment hospitalisation, resulting in missing information for that region.

Children residing in Southern Italy had the lowest proportion of patients managed within their region of residence: 72% for diagnosis, 58% for surgery, and 69% for chemotherapy. Radiotherapy was delivered within the in only 38% of the cases.

### 3.3. Survival and Progression

**Figure 1:**
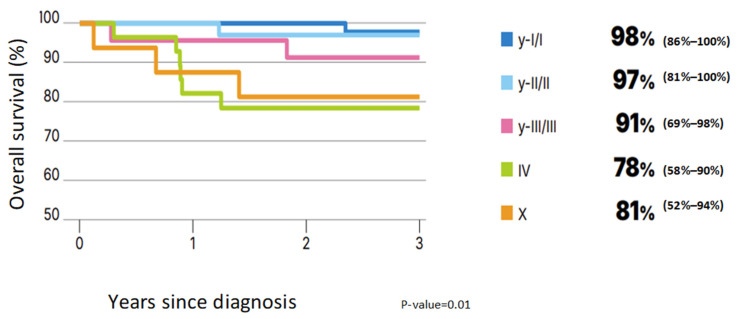
Wilms tumour in Italy: 3-year-survival by stage at diagnosis.

Three-year survival according to tier 2 stage at diagnosis decreased with the increasing stage. Figure 1 reports 3-year survival estimates by stage. Survival was highest for Stage I (97.8%, 95%CI: 86–100%) and Stage II (97.1%, 95%CI: 81–100%), with decreasing survival for Stage III (91.3%, 95%CI: 69–98%), Stage IV (78.4% 95%CI: 58–90%), and unknown stage (81.3%, 95%CI: 52–94%). The log-rank test for survival by stage provided strong evidence against the null hypothesis of equality of the survivor function across stages (*p*-value= 0.01).

**Figure 2:**
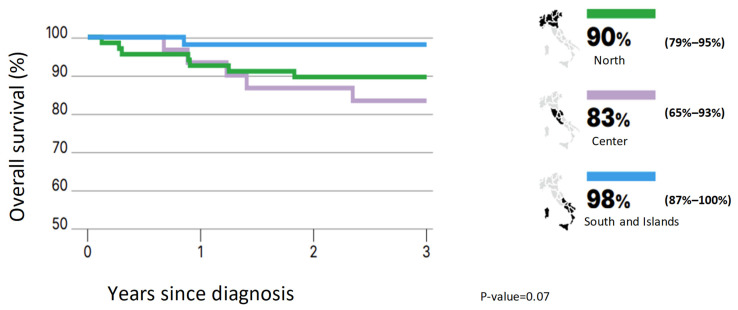
Wilms tumour in Italy: 3-year survival by area.

Figure 2 displays survival curves by macro-area over time. The highest 3-year survival was observed in the south (98.0%, 95%CI: 87–100%), followed by the north (89.5%, 95%CI: 79–95%) and centre (83.3%, 95%CI: 65–93%) (Supplementary Table S4). A total of 17 patients died during follow-up. Among cases with available information on cause of death (64%, N = 11), all deaths were attributed to WT. For six patients, the cause of death was not reported (Table 1).

In the multivariable model reported in Supplementary Table S5, Stage IV was associated with poorer survival compared with Stage I. Patients from the south appeared to have better survival than those from the north. Similarly, individuals who received treatment outside their region of residence showed better survival compared with those who did not migrate for care. However, it is important to note that WT is a rare cancer and the number of fatal events was very low. This resulted in wide confidence intervals for hazard ratios across age groups, geographic areas, stages, and migration status, indicating substantial uncertainty around these estimates, and suggesting that the true effects may vary considerably.

Information on relapse or progression was available for 106 of the 148 patients (72%) (Table 1). Among these patients, 21% experienced disease progression or relapse, with a mean time to progression of 311 days. Nearly all patients with progression (all but four) had Stage II or higher at diagnosis. Ten of the patients with documented progression died from the cancer within three years of diagnosis.

## 4. Discussion

Cancer registries are essential tools for quantifying and describing the incidence of malignancies, providing a robust foundation for research, clinical practice, and public health planning. This is the first paper providing population-based estimates of the distribution of stage at diagnosis and survival by stage for WT in Italy.

The PBCRs were able to collect stage at diagnosis for 92% of cases using the simplest classification (Tier 1), and for 82% of cases using the more detailed scheme (Tier 2).

We observed a marked survival gap at three years after diagnosis between Stage I and Stage IV patients (98% versus 78%). The proportions of Tier 2 Stage III and IV cases were similar in the north and south; the centre contributed only Tier 1 stage data because one regional registry was unable to collect Tier 2 variables. Likewise, the distribution of Tier 1 metastatic stage (M) was comparable across areas.

However, in line with previous analyses [18], geographical disparities in survival persist in Italy (even if not significant), with a 15-percentage point difference between the highest and lowest three-year survival estimates. Nationally, cumulative five-year survival for WT cases increased from 87% in 1998–2002 [18] to 90% in 2013–2017 [8]. The AIEOP Wilms Tumor Working Group reported a five-year event-free survival of 84% and OS of 92% for unilateral WT treated with the TW 2003 regimen, while OS decreased to 73% for anaplastic WT [19] therefore confirming that anaplasia, like stage at diagnosis, remains strongly associated with poorer prognosis [20,21].

In our study, information on anaplasia was available for 70% of the cases, of which 15% were classified as positive. Considering that the assessment of anaplasia is routinely performed due to its clinical importance, and based on discussions with clinical experts involved in the project, it is plausible that a substantial proportion of the missing data corresponds to ‘anaplasia not present’ as this feature is often omitted from pathological report when negative. Under this assumption, the resulting proportion of anaplasia presence—around 11%—would be more consistent with European figures [22]. However, this assumption represents a potential limitation of the study, as the true status of cases with missing information cannot be definitively ascertained.

The companion BENCHISTA international project reported a 3-year survival of 95% for WT across European countries, based on 1817 cases [23], slightly higher than the estimate observed in the Italian BENCHISTA cohort (91%, Supplementary Table S4). Notably, a substantial gap emerged when examining children classified as M+ (Tier 2): survival at 3 years reached 87% in Europe versus 78% in Italy. Furthermore, the proportion of Stage IV cases in our study (22%) was similar to the proportion observed in the UK and Ireland, which reported the highest proportion of M+ cases in the areas investigated by the international study. Three-year survival for M+ patients ranged from 100% in Northern Europe to 79% in Eastern Europe.

If the search for distant metastases—particularly lung evaluation—was conducted consistently across European centres, these findings suggest that there remains considerable space for improvement in the management of children presenting with metastatic disease and in predicting tumour recurrence in Italy, as well as in other countries [24,25,26,27]. The key priority is to enhance treatment strategies for advanced-stage cases.

We have recently begun to understand the molecular mechanisms underlying relapse in patients with WT [28] and the mechanisms underlying chemotherapy resistance in children with Wilms tumours with favourable histology [29]. New therapies are being studied for patients with reduced responsiveness to conventional chemotherapy.

With the recent advent of novel therapies, such as immune checkpoint inhibitors, CAR-T cell therapy, and WT-1-targeted cancer vaccines [30,31,32], which are radically transforming treatment paradigms and long-term outcomes, closer collaboration between paediatric oncologists and epidemiologists is vital. Such partnership will be increasingly essential to evaluate the true impact of the advancements on the prognosis of these rare tumours at the population level.

To improve outcomes for Stage IV or relapsed Wilms tumour, it is crucial that treatment is planned by an experienced multidisciplinary team composed of paediatric oncologists, paediatric surgeons, paediatric radiation oncologists, and a pathologist with experience on histopathological finding for WT [33,34]. Induced chemotherapy modifications, in case of nephrectomy samples obtained after treatment, and the presence of focal or diffuse anaplasia, can negatively influence the prognosis [35]. Furthermore, the presence of specific genetic abnormalities should always be sought and could be reported in analyses performed on population tumour registry data to better evaluate overall survival [36,37,38,39].

Paediatric oncology care in Italy has historically developed as a coordinated national network rather than a fully centralized system. Since the late 1970s, the AIEOP has played a central role in organizing and harmonizing the diagnosis and treatment of childhood cancers, including Wilms tumour, across the country. Within this model, multiple paediatric oncology centres are geographically distributed and accredited to participate in shared national and international standard treatment protocols and collaborative studies, with centralized data collection, common risk stratification criteria, and collaborative clinical governance. While some centres have progressively developed higher patient volumes and greater expertise, particularly for rare or complex cases, the AIEOP network has maintained a balance between specialization and territorial accessibility of care. Referral pathways are therefore flexible and primarily guided by clinical complexity, local expertise, and family choice, rather than by formal national centralization. This network-based organization provides an important framework for interpreting the regional distribution of treatments and patterns of inter-regional patient migration observed in the present analysis and distinguishes the Italian model from more centralized systems adopted in other countries.

Within this framework, our study provides national migration patterns for diagnosis and treatment. We observed a high percentage of in-region diagnoses, indicating good territorial coverage for initial assessments. The proportion of patients traveling outside the region was higher for surgery and radiotherapy, underscoring the importance of performing these treatments in specialized hospitals. With the only exception of the south, the great majority of patients received diagnosis and treatment in the same area of residence (Table 3). Surgery and, especially, radiotherapy still represent the causes of healthcare migration to Northern Italy, where the expectation of receiving better care remains common, although this is not reflected in paediatric renal tumour outcomes.

Although high-volume centres were more commonly located in Northern and Central Italy, outcomes in the south did not appear to be affected. This represents a very positive finding from a population-based study on childhood cancers, highlighting the overall effectiveness of the Italian National Health System, supported by the primary care paediatricians and the specialized AIEOP clinical network [40].

These results suggest that, although health migration from Southern Italy to other regions was reported during the same period [41], this phenomenon does not seem to affect patient survival. Migration for surgical treatment or radiotherapy to hospitals outside the region of residence indicates, on the one hand, a lack of referral facilities specialized in the comprehensive management of WT, but on the other hand, it demonstrates the presence of a collaborative network among paediatric oncology centres in Italy that supports patients throughout their care pathway.

We did not observe a clear difference in outcomes between patients who migrated and those who did not; although the estimated hazard ratio suggested a potential reduction in risk among patients who migrated (HR = 0.6, not significant). These findings are based on a limited number of events and should therefore be considered exploratory. In the Italian setting, patients who migrate are typically treated in AIEOP high-volume centres, which may contribute to the observed pattern [42].

In the south, high survival rates have been observed both among patients treated within their own region and among those who travel elsewhere for part of their treatment. Overall survival in the South is likely slightly higher because all patients are followed by an AIEOP centre—or by hospitals closely connected to one—which may ensure more stable and continuous care. The patient remains linked to the centre that initially took charge of their care, which was also likely the one able to refer them to another centre when the planned treatment could not be performed locally.

However, this network cannot replace the essential involvement of the entire family, the local community, and the child’s daily routines, nor it can compensate for the prolonged absences of family members from work and the direct and indirect costs associated with health migration. Efforts must be made by public health institutions to make the distribution of equipped paediatric oncology centres in Italy more homogeneous.

Among the cases collected from PBCRs, 125 out of 148 were successfully matched with those reported in model 1.01 of the AIEOP platform, reducing the number of cases for which staging could not be assigned based on PBCR information. The data-linkage from the PBCRs involved in the project with the AIEOP clinical databases showed the possibility of mutual enrichment. The acquisition of better completeness of stage at diagnosis could result in better study of geographic variations in survival. Conversely, given the high percentages of long-surviving patients achieved with this tumour, PBCR data will be very useful to clinicians in the long-term follow-up of patients when they leave the observation period of the protocols or move from the place of care. Valid and codified staging at diagnosis will become increasingly useful in understanding any late effects, second tumours, or long-term recurrences.

Finally, because AIEOP relies on cases reported directly by clinical centres, whereas PBCRs systematically search and verify all new diagnoses within the population, the enhanced completeness of incidence achieved through this collaboration represents a major added value.

### Strengths and Weaknesses

This study showed that adopting the TG allows for a more accurate characterization of childhood cancers. However, its implementation is not straightforward, particularly for general PBCRs, even though childhood cancers are rare. The main challenge relates to the GDPR, which is often interpreted in a highly restrictive manner at the regional level, limiting the collection of detailed clinical variables. Nevertheless, the systematic collection of these data should be strongly encouraged.

The international BENCHISTA project demonstrated that stage at diagnosis partly explains the wide European disparities in survival across countries [9,23]. Treatment is likely to play an even more important role than stage at presentation, and survival analyses stratified by stage help identify for which groups treatment remains particularly challenging. In our study, the main difficulties appear to concern the management of patients presenting with distant metastases.

The low percentage of Tier 2 stage, mainly in Central Italy, was due to the difficulties in receiving the clinical report from the hospital of admission. Sharing of clinical document/data remains a criticism in certain Italian regions because of the wrong interpretation of the European GDPR.

The national coverage of PBCRs in Italy is still incomplete, and it remains essential to work toward a unified national registry, including a dedicated function for childhood cancers, while preserving the opportunity for internal geographic comparisons. Achieving this goal will require strong collaboration between hospitals and PBCRs. In this regard, a formal agreement between the two relevant associations, AIEOP and AIRTUM, is crucial. Clear guidelines on the interpretation of the European GDPR are also urgently needed to facilitate data sharing and interoperability, while ensuring harmonized practices across regions [43].

Another limitation of the study is the relatively short follow-up. The second phase of the BENCHISTA project will extend follow-up to five years, enabling a more complete collection of clinical variables and improving the overall completeness and quality of incidence data.

## 5. Conclusions

This study highlights the TG as a valuable tool for standardizing the collection of clinical variables, particularly tumour stage at diagnosis, which should be systematically collected and made available at the population level. The findings also underline the importance of continued monitoring of outcomes at the population level.

From a public health and organizational perspective, our results point to the need to strengthen the collection of key clinical information and to improve collaboration between clinicians and epidemiologists. This could be supported by a national framework to facilitate data sharing and enable routine linkage between clinical and cancer registry networks.

In Italy, challenges persist in the management of metastatic cases. In addition, patient pathways indicate that surgery and especially radiotherapy remain important drivers of healthcare migration from Southern to Northern regions.

Overall, these findings contribute to the European effort to benchmark paediatric cancer care and identify opportunities to improve both clinical practice and healthcare system organization. Strengthening timely and comprehensive data collection will be essential to ensure full national registry coverage and ultimately improve outcomes in paediatric oncology.

## Figures and Tables

**Table 1:** Wilms tumour in Italy: number of cases, demographic and tumours characteristics, treatment, relapse, cause of death (CoD) and lost to follow-up by macro-area. Period of diagnosis 2013–2017.

	Macro-Areas			
	North ^	Centre *	South and Islands °	Total
No. of cases (%)	67 (45%)	30 (21%)	51 (34%)	148 (100%)
Mean age at diagnosis in months	44	33	48	44
% <1 year of age	17%	20%	20%	18%
% 1–4 years	55%	63%	51%	56%
% 5–14 years	28%	17%	29%	26%
M/F ratio	0.86	0.76	1.08	0.91
% MV	97%	97%	96%	97%
% Laterality available	100%	77%	90%	92%
% Anaplasia investigated (% anaplasia present)	84% (16%)	33% (30%)	73% (11%)	70% (15%)
% Chemotherapy prior to surgery (SIOP)	69%	53%	55%	61%
% Surgery + chemotherapy (COG)	30%	43%	45%	38%
% Relapse/recurrence available (% relapse/recurrence)	75% (28%)	50% (6%)	80% (17%)	72% (21%)
% COD available (% death due to cancer)	88% (100%)	33% (100%)	67% (100%)	64% (100%)
% Lost to follow-up/censored	3%	0%	6%	3%

* = Marche, Toscana, Umbria, Lazio; ^ = Bergamo, Insubria Varese Como, Mantova e Cremona, Emilia Romagna, Friuli Venezia Giulia, Genova, Milano, Monza e Brianza, Piemonte, Trento, Veneto; ° = Puglia, Basilicata, Catania-Messina-Siracusa-Enna, Nord Sardegna, Nuoro, Palermo, Ragusa e Caltanissetta, Siracusa, Trapani, Campania; MV = microscopically verified cases.

**Table 2:** Wilms tumour in Italy: distribution by stage at diagnosis (Tier 1 and 2) and by area.

Macro-Area	Wilms Tumour Stage Tier 1				Wilms Tumour Stage Tier 2					
	L	M	X	Total	y-I/I	y-II/II	y-III/III	IV	X	Total
North	51	15	1	67	24	15	11	15	2	67
*%*	*76.1*	*22.4*	*1.5*	*100*	*35.8*	*22.4*	*16.4*	*22.4*	*3.0*	*100*
Centre	24	6	0	30	10	5	3	2	10	30
*%*	*80.0*	*20.0*	*0.0*	*100*	*33.3*	*16.7*	*6.7*	*6.7*	*33.3*	*100*
South and Islands	39	11	1	51	13	14	9	11	4	51
*%*	*76.5*	*21.6*	*2.0*	*100*	*25.5*	*27.5*	*17.7*	*21.6*	*7.8*	*100*
BENCHSTA-ITA	114	32	2	148	47	34	23	28	16	148
*%*	*77.0*	*21.6*	*1.4*	*100*	*31.8*	*23.0*	*15.5*	*18.9*	*10.8*	*100*

Fisher’s exact: Pr = 0.7, excluding the missing cases.

**Table 3:** Wilms tumour in Italy: Number of cases, number of patients with missing information on hospital(H) and proportion of patients diagnosed and treated within their region of residence (by treatment type) by macro-area.

Macro-Area	Diagnosis			Surgery			Chemotherapy			Radiotherapy		
	No. of Cases	H Unknown	H in Region %	No. Treated with Surgery	H Unknown	H in Region %	No. Treated with Chemo	H Unknown	H in Region %	No. Treated with Radio	H Unknown	H in Region %
North	67	3	92%	63	1	90%	62	1	90%	20	0	95%
Centre	18	0	100%	18	0	94%	17	0	94%	2	0	100%
South and Islands	51	1	72%	51	1	58%	50	1	69%	16	1	38%
BENCHSTA-ITA	136	4	83%	132	2	77%	129	2	81%	38	1	71%

Tuscany was excluded due to unavailability of hospital information.

## Data Availability

Individual-level data is unavailable due to privacy or ethical restrictions.

## Supplementary Materials

The following supporting information can be downloaded at: https://www.mdpi.com/article/10.3390/cancers18132111/s1, Table S1: Staging system for Wilms tumour based on the Toronto Childhood Cancer Stage Guidelines (ref. [16] by Aitken et al.) according to the protocol (a) COG (b) SIOP; Table S2: Wilms tumour: Non-stage prognostic factor (i.e., Anaplasia) by Area; Table S3: Wilms tumour: Treatment combination distribution (not in temporal order) by Area; Table S4: Wilms tumour in Italy: 3-year-survival by stage at diagnosis and by Area; Table S5: Wilms tumour in Italy: multivariate Cox-proportional hazard models adjusted by stage at diagnosis, age class, area and migration.

## Appendix A

BENCHISTA-ITA WG: Basilicata Cancer Registry, IRCCS CROB: Rocco Galasso. Bergamo Cancer Registry: Giuseppe Sampietro. Campania Childhood Cancer Registry: Marcella Sessa *, Patrizia Piga. Childhood Cancer Registry of Piedmont: Milena M Maule, Carlotta Sacerdote *. Cremona & Mantova Cancer Registry: Paola Ballotari. Friuli-Venezia Giulia Cancer Registry, CRO Aviano National Cancer Institute: Luigino Dal Maso Integrated Cancer Registry CT-ME-EN: Rosalia Ragusa, Antonina Torrisi. Liguria Cancer Registry, Ospedale Policlinico San Martino IRCCS: Claudia Casella, Antonella Puppo. Monza-Brianza Cancer Registry: Magda Rognoni. Palermo Province Cancer Registry: Rosalba Amodio. Puglia Cancer Registry: Francesco Cuccaro, Danila Bruno. Registro Tumori ATS Della Città metropolitana di Milano: Antonio G Russo, Federico Gervasi. Registro Tumori ATS Insubria: Maria L Gambino. Registro Tumori dell’Emilia-Romagna, Unità di Piacenza: Pietro Seghini. Registro Tumori dell’Emilia-Romagna, Unità di Parma: Maria Michiara. Registro Tumori dell’Emilia-Romagna, Unità di Reggio Emilia: Lucia Mangone. Registro Tumori dell’Emilia-Romagna, Unità di Modena: Giuliano Carrozzi. Registro Tumori dell’Emilia-Romagna, Unità di Ferrara: Caterina Palmonari. Registro Tumori dell’Emilia-Romagna, Unità Della Romagna, IRCCS IRST Meldola: Silvia Mancini. Registro Tumori di Ragusa e Caltanissetta: Eugenia Spata. Registro Tumori Regione Marche: Sonia Manasse, Paola Coccia. Registro Tumori Umbria: Fabrizio Stracci. Registro Tumori Sassari: Daniela Piras. Registro Tumori Lazio: Ilaria Cozzi, Enrica Santelli. Registro Tumori Nuoro: Pasquala Pinna. Siracusa Cancer Registry: Francesca Bella. Toscana Cancer Registry: Adele Caldarella, Teresa Intrieri. Registro Tumori Trapani_Agrigento, Dipartimento di Prevenzione, ASP Trapani: Tiziana Scuderi. Trento Cancer Registry (Trento CR), Servizio Epidemiologia Clinica e Valutativa, APSS Trento: William Mantovani. Veneto Cancer Registry, Regional Epidemiological Service, Azienda Zero, Padova, Italy: Manuel Zorzi, Stefano Guzzinati.

Project Management Team (PMT): Riccardo Capocaccia, Epidemiologia e Prevenzione, Indipendent Advisory Board, Milan, Italy; Massimo Conte, Clinical Oncology Unit, IRCCS Istituto Giannina Gaslini, Genoa, Italy; Fabio Savoia, Campania Childhood Cancer Registry, Naples, Italy.

Clinical Lead Experts/Other Representatives: Andrea Di Cataldo: Università di Catania, Catania, Italy; Paolo D’Angelo: Ospedali Civico di Cristina Benfratelli di Palermo; Andrea Pession: IRCCS Azienda Ospedaliero-Universitaria di Bologna, Bologna, Italy; Giulia Stabile, CINECA Bologna; Riccardo Haupt, Maria Luisa Garrè: IRCCS Istituto Giannina Gaslini, Genoa; Gianni Virgili: University of Florence, Florence, Italy; Andrea Tittarelli, Claudio Tresoldi, Giovanna Tagliabue: Department of Predictive Medicine and Prevention at the National Cancer Institute of Milan, Italy. * = Project Management Team member.
